# *Notes from the Field:* Conjunctivitis Caused by Toxigenic *Corynebacterium ulcerans* — Missouri, 2018

**DOI:** 10.15585/mmwr.mm6827a3

**Published:** 2019-07-12

**Authors:** Lauren M. Weil, Cindy Butler, Karla R. Howell, Sarah Sharr, Grace L. Paley, Andrew J. W. Huang, Robi N. Maamari, Lucia C. Pawloski, Pamela K. Cassiday, Anna M. Acosta, Susan Hariri, Tejpratap S. P. Tiwari

**Affiliations:** ^1^Epidemic Intelligence Service, CDC; ^2^Division of Bacterial Diseases, National Center for Immunization and Respiratory Diseases, CDC; ^3^Missouri Department of Health and Senior Services, Jefferson City, Missouri; ^4^St. Louis County Department of Public Health, St. Louis, Missouri; ^5^Missouri State Public Health Laboratory, Jefferson City, Missouri; ^6^Department of Ophthalmology and Visual Sciences, Washington University School of Medicine, St. Louis, Missouri.

On December 12, 2018, an immunocompromised man with non-Hodgkin’s lymphoma, aged 73 years, was evaluated by an ophthalmologist for left eyelid redness, swelling, and eye discharge and received a diagnosis of ligneous (pseudomembranous) conjunctivitis. The pseudomembrane was debrided and sent for culture, and the patient was prescribed oral amoxicillin clavulanate and moxifloxacin eye drops, with topical loteprednol and cyclosporine to decrease the robust inflammatory response. *Corynebacterium ulcerans*, one of three species of Corynebacterium (in addition to *C. diphtheriae *and* C. psuedotuberculosis*) that can harbor the diphtheria toxin–producing gene was initially identified by matrix-assisted laser desorption ionization time-of-flight mass spectrometry performed on an isolate obtained from culture of the pseudomembrane at a Missouri hospital on December 13. The Missouri Department of Health and Senior Services (MDHSS) laboratory-confirmed *C. ulcerans* by culture and forwarded the isolate to CDC for toxin testing. On December 28, CDC confirmed toxin-producing *C. ulcerans*. The patient had no systemic symptoms, was not hospitalized, and did not receive diphtheria antitoxin. On January 11, 2019, following multiple membrane removals and no residual membrane; cultures of conjunctival swabs tested by the hospital were negative for *C. ulcerans*. The patient was not up-to-date for tetanus-diphtheria (Td) vaccine and had postponed vaccination because of his ongoing cancer treatment.

Case investigation by MDHSS and the St. Louis County Department of Public Health identified one household contact. Paired nasal and throat swabs collected from the patient (posttreatment) and the household contact to assess carriage were negative by culture for *C. ulcerans*. The household contact was not offered antibiotic postexposure prophylaxis (PEP) and declined a Td booster. Ophthalmology staff members who had direct contact with the patient reported wearing recommended personal protective equipment and declined PEP. No identified contacts developed disease. The patient lived with two dogs; neither was reported to be ill or examined by a veterinarian, and the patient declined to have the dogs tested for *C. ulcerans*.

Toxigenic strains of *Corynebacterium diphtheriae* are transmitted person-to-person and cause respiratory and cutaneous diphtheria; infections of other mucous membranes, such as the eye, have been reported (*1*). This is the first reported case of conjunctivitis caused by a toxigenic strain of *C. ulcerans*, which, along with *C. pseudotuberculosis*, is a zoonotic species. Toxigenic *C. ulcerans* has been isolated from a variety of animal species. Infected livestock and pets such as dogs and cats are recognized sources of occasional *C. ulcerans* infection in humans, particularly in older adults who are either unvaccinated with diphtheria toxoid–containing vaccines or have not received recommended booster doses. Since the late 1980s, *C. ulcerans* has been increasingly reported as a cause of respiratory and cutaneous diphtheria-like illness. Although it is possible, secondary person-to-person transmission of *C. ulcerans* has not been verified.

Because diphtheria toxoid–containing vaccines target diphtheria toxin, vaccination with these vaccines most likely prevents toxin-mediated disease caused by all toxigenic strains of Corynebacterium. Because there are common exposures among household contacts and person-to-person transmission might be possible, vaccination status of household and other close contacts (e.g., medical providers) should be assessed, and contacts who are not up-to-date should be offered vaccination (*2*,*3*).

Antibiotic treatment of illnesses caused by toxin-producing *C. ulcerans* should follow treatment guidelines for patients infected with *C. diphtheriae* (*1*). Diphtheria antitoxin is recommended for respiratory infections caused by toxigenic *C. diphtheriae* or *C. ulcerans* (*4*). Health care providers should be aware that *C. ulcerans* infection can be acquired from pets, particularly by elderly or immunocompromised persons (*3*,*5*). Both *C. diphtheriae* and *C. ulcerans* can become toxigenic through lysogeny by beta-corynebacteriophages harboring the diphtheria toxin gene. Circulation of toxigenic *C. ulcerans* in animals highlights an animal reservoir for corynebacteriophages. This poses programmatic challenges to eradicating diphtheria caused by toxigenic *C. diphtheriae* and emphasizes the need to maintain high human population immunity through diphtheria vaccination, including recommended decennial booster doses (*6*).
